# Missing Oral Health-Related Data in the interRAI-HC - Associations with Selected Variables of General Health and the Effect of Multiple Imputation on the Relationship between Oral and General Health

**DOI:** 10.1371/journal.pone.0146065

**Published:** 2015-12-30

**Authors:** Stefanie Krausch-Hofmann, Kris Bogaerts, Michael Hofmann, Johanna de Almeida Mello, Nádia Cristina Fávaro Moreira, Emmanuel Lesaffre, Dominique Declerck, Anja Declercq, Joke Duyck

**Affiliations:** 1 Department of Oral Health Sciences, Population Studies in Oral Health, KU Leuven, Leuven, Belgium; 2 Department of Public Health and Primary Care, KU Leuven and Hasselt University I-BioStat, KU Leuven, Leuven, Belgium; 3 Department of Neurosciences, Research Group Experimental Oto-Rhino-Laryngology, KU Leuven, Leuven, Belgium; 4 LUCAS, Center for Care Research and Consultancy, KU Leuven, Leuven, Belgium; 5 Department of Oral Health Sciences, Biomaterials, KU Leuven, Leuven, Belgium; University of Brescia, ITALY

## Abstract

**Background:**

Missing data within the comprehensive geriatric assessment of the interRAI suite of assessment instruments potentially imply the under-detection of conditions that require care as well as the risk of biased statistical results. Impaired oral health in older individuals has to be registered accurately as it causes pain and discomfort and is related to the general health status.

**Objective:**

This study was based on interRAI-Home Care (HC) baseline data from 7590 subjects (mean age 81.2 years, SD 6.9) in Belgium. It was investigated if missingness of the oral health-related items was associated with selected variables of general health. It was also determined if multiple imputation of missing data affected the associations between oral and general health.

**Materials and Methods:**

Multivariable logistic regression was used to determine if the prevalence of missingness in the oral health-related variables was associated with activities of daily life (ADLH), cognitive performance (CPS2) and depression (DRS). Associations between oral health and ADLH, CPS2 and DRS were determined, with missing data treated by 1. the complete-case technique and 2. by multiple imputation, and results were compared.

**Results:**

The individual oral health-related variables had a similar proportion of missing values, ranging from 16.3% to 17.2%. The prevalence of missing data in all oral health-related variables was significantly associated with symptoms of depression (dental prosthesis use OR 1.66, CI 1.41–1.95; damaged teeth OR 1.74, CI 1.48–2.04; chewing problems OR 1.74, CI 1.47–2.05; dry mouth OR 1.65, CI 1.40–1.94). Missingness in damaged teeth (OR 1.27, CI 1.08–1.48), chewing problems (OR 1.22, CI 1.04–1.44) and dry mouth (OR 1.23, CI 1.05–1.44) occurred more frequently in cognitively impaired subjects. ADLH was not associated with the prevalence of missing data. When comparing the complete-case technique with the multiple imputation approach, nearly identical odds ratios characterized the associations between oral and general health.

**Conclusion:**

Cognitively impaired and depressive individuals had a higher risk of missing oral health-related information. Associations between oral health and ADLH, CPS2 and DRS were not influenced by multiple imputation of missing data. Further research should concentrate on the mechanisms that mediate the occurrence of missingness to develop preventative strategies.

## Introduction

The interRAI suite of instruments consists of third-generation comprehensive geriatric assessment tools, designed to define what kind of care is required for an individual care-dependent person [1]. Based on the multidimensional and multi-domain computer-assisted assessment, possible prevention and treatment options as well as referral for further evaluation is considered. In addition, interRAI data can be a source for quality-of-care evaluations, informed health policy decisions and research. In the context of this broad scope of utilizations, quality of interRAI data needs to be examined.

Missing values–defined as incompleteness of the registered data—can occur due to technical disturbances, if subjects refuse or are unable to respond, or if assessors do not fill in the instrument thoroughly. If the distribution of missingness is not completely at random, results based on this data might be biased if not appropriately analyzed [2]. Missing data within the interRAI also implies the potential under-detection of medical, psychological or social conditions that require care.

In a multi-center study based on the interRAI—Acute Care in Belgium, Wellens et al. (2011) found a substantial number of missing data [3]. Vanneste et al. (2015) described missing data rates equal or more than 15% for the Belgian interRAI-Home Care (HC) and concluded that further evaluation is necessary [4].

This research article focuses on missingness in the oral health-related variables of the interRAI-HC. A thorough and accurate registration of oral health is essential as oral infections are related to pain and discomfort. They are also the main cause for tooth loss [5], which may in turn affect chewing function and nutrition [6]. Additionally, oral health in older individuals is related to a number of systemic diseases, physical and cognitive status, depression, institutionalization and mortality [7–13].

In the present study, an interRAI-HC dataset of older Belgian home-care patients was used to investigate the relationship between missing values of the oral health-related variables and selected variables of general health. It was hypothesized that missing data of the oral health-related variables do occur more frequently in subjects with a compromised general health status. Furthermore, associations between oral health and selected variables of general health were analyzed before and after multiple imputation of the missing data. Assuming that missingness is related to poor general health, it was hypothesized that associations between oral and general health were more pronounced after replacing missing data by estimated values using the multiple imputation method.

## Materials and Methods

### Dataset and ethical considerations

The present study used 01/2010-08/2013 baseline data from the Protocol 3 project that included frail older people (65+ years) receiving home care in Belgium. The original project intended to identify interventions that delay institutionalization. The Belgian version of the interRAI-HC was applied by professional care givers who received three days of training. Detailed information of the study design was described elsewhere [14].

The protocol 3 project was approved by the Belgian Privacy Commission and by the Ethics committee of the Belgian Universities—Université Catholique de Louvain and Katholieke Universiteit Leuven with dossier number B40320108337. Approval included the retrospective study that is presented here. Older persons were asked to sign an informed consent agreement. In case they were not capable of signing this document, a family member or another legal representative signed it on their behalf, as stipulated by Belgian law. Clients had the right not to participate in the research and they could withdraw their consent at any time. In that case, all data for this person was removed. All data were anonymized and de-identified before the dataset was sent to the researchers for analysis.

### Oral health and selected variables of general health

Section K of the BelRAI-HC contains 4 dichotomous (yes/no) oral health-related questions. It registers *dental prosthesis use* and the following oral health-related problems: *cracked*, *broken*, *not intact teeth (damaged teeth)*, *chewing difficulty* and *dry mouth*—occurred in the three days prior to the assessment. The data were collected by interview and observation of the subject, as well as inspection of the mouth.

General health was operationalized by three scales that represent a quantified summary of the subject's functional, cognitive and mental condition. Each scale is calculated automatically by the interRAI algorithms, based on a number of individual items.

The validated Activities of Daily Life Hierarchy Scale (ADLH) represents the functional ability of the subject to perform the following self-care tasks: personal hygiene, toilet use, locomotion, and eating. The resulting 7-point ADLH scale ranges from 0 (completely independent) to 6 (completely dependent) [15]. The second version of the internationally validated Cognitive Performance Scale (CPS2) was used to operationalize the cognitive status of the subject. It is based on a variety of items concerning cognitive skills for everyday decision making, short-term memory, procedural memory, self-expression and eating. The 7-point scale ranges from 0 (intact) to 6 (severely cognitively impaired) [16–17]. The Depression Rating Scale (DRS) was used to represent the mental condition of the subject with reference to symptoms of depression during the last three days. The DRS is based on a number of items as negative statements, expression of unrealistic fears, repetitive complaints or crying for no reason. The scores range from 0 to 14 with higher scores representing more pronounced symptoms of depression [18].

### Statistical analyses

ADLH, CPS2 and DRS were recoded into dichotomous variables using validated cut-off points. Subjects with a ADLH score ≥3 were considered as functionally impaired [19] and cognitive impairment was denoted for scores ≥2 on the CPS2 Scale [17, 20]. For the Depression Rating Scale, scores ≥3 were used to characterize an older person as being depressed [18]. Dichotomization of the scales was necessary due to the distribution characteristics of these variables and the requirements of the statistical procedures used.

For each oral health-related item, a missing-value indicator was created and associations with ADLH, CPS2, and DRS were evaluated. Subsequently, associations between the observed values of the oral health-related variables and ADLH, CPS2, and DRS were analyzed for subjects with completely registered observations (complete-case technique). After multiple imputation of missing values, associations between oral health and ADLH, CPS2, and DRS were re-evaluated and results were compared. Age and gender were examined as potential confounding variables.

For all analyses, multivariable logistic regression included those predictors and covariates that were significantly associated with the outcome variable on a p<0.05 level of significance in univariable logistic regression.

Multiple imputations were created using the fully conditional specification method [21], also referred to as MICE (Multivariate Imputation by Chained Equations). All outcome variables, predictors, and covariates were used in the imputation model. With the exception of age for which a linear regression model was applied, variables were imputed by a logistic regression model. Ten imputations were performed and results were summarized over the 10 imputed data sets using PROC MIANALYZE.

Multicollinearity of the predictors was examined by Spearman correlations and Variance Inflation Factors (VIF). The correlation matrix revealed low Spearman correlation coefficients (<0.3). Using the 2.5 criterion for VIF, it was concluded that multicollinearity had not to be considered a problem for the multivariable analysis.

Analyses were performed by using the statistical software SAS, version 9.3 (SAS Institute, Inc., Cary, NC).

## Results

### Description of the dataset

InterRAI-HC baseline assessments were available for 8123 individuals. As the original project intended to include individuals aged 65 or older, subjects with a chronological age less than 65 years were removed. Cases with undefined dates of measurement or birth were also excluded. After cleaning of the dataset, 7590 subjects were available for analysis. The mean age was 81.2 years (SD 6.9, range 65–102) and 69% (5203) were female.

Table 1 presents the descriptive statistics and illustrates that the individual oral health-related variables had a similar proportion of missing values, ranging from 16.31% to 17.18%.

**Table 1:** Descriptive statistics of oral health-related variables and corresponding missingness.

	N	% Missingness	% Yes, Complete-case technique	% Yes, Multiple imputation
Number of patients	7590			
Dental prosthesis	6306	16.92	74.15	73.96
Damaged teeth	6286	17.18	13.95	14.23
Chewing problems	6352	16.31	12.52	12.72
Dry mouth	6326	16.65	17.47	17.57

Using the completely registered observations, about 74.15% of the subjects wore a *dental prosthesis*. Damaged *teeth*, *chewing problems* and *dry mouth* were registered for 13.95%, 12.52% and 17.47% of the subjects, respectively. After multiple imputation of the incomplete observations, the percentage of subjects with a *dental prosthesis* decreased to 73.96%. The prevalence of d*amaged teeth*, *chewing problems* and *dry mouth* increased to 14.23%, 12.72% and 17.57%, respectively.

General health variables were distributed as follows: Assessed by the ADLH scale, 50.1% of the participants were functionally dependent with 8.1% missing values. Cognitive impairment (CPS2) was detected for 43.9% with a missing value proportion of 8.3%. The DRS classified 29.6% of the participants as *depressed* with 7.5% missing values.

### Associations between oral health-related variables, their corresponding missingness and ADLH, CPS2, DRS

Table 2 shows odd's ratios (OR) and their 95% confidence intervals (CI) to illustrate associations between oral health-related items, their corresponding missingness, ADLH, CPS2, DRS and covariates. In the following, results are described for the multivariable analyses.

**Table 2:** Oral health-related variables and corresponding missing values: associations with ADLH, CPS2 and DRS.

	Oral health-related variables, Odds ratio [95% Confidence interval]					
	Missing values		Complete Case Technique		With Multiple Imutation	
	Univariable	Multivariable	Univariable	Multivariable	Univariable	Multivariable
Dental prosthesis						
ADLH (≥3 vs <3)	0.98 [0.84–1.13]		** 0.83 [0.74–0.93]	** 0.82 [0.73–0.92]	** 0.82 [0.73–0.92]	*** 0.80 [0.70–0.90]
CPS2 (≥2 vs <2)	*** 1.31 [1.13–1.52]	1.15 [0.98–1.34]	* 0.89 [0.79–1.00]	0.93 [0.82–1.05]	0.90 [0.81–1.01]	
DRS (≥3 vs <3)	*** 1.93 [1.67–2.23]	*** 1.66 [1.41–1.95]	0.90 [0.80–1.02]		0.92 [0.81–1.04]	
Gender (m vs f)	1.13 [1.00–1.29]		*** 0.68 [0.61–0.77]	*** 0.73 [0.64–0.83]	*** 0.67 [0.59–0.76]	*** 0.71 [0.62–0.81]
Age (years)	1.00 [0.99–1.01]		*** 1.05 [1.04–1.06]	*** 1.05 [1.04–1.06]	*** 1.05 [1.04–1.06]	*** 1.05 [1.04–1.06]
Damaged teeth						
ADLH (≥3 vs <3)	0.97 [0.84–1.12]		1.13 [0.98–1.31]		1.14 [0.98–1.31]	
CPS2(≥2 vs <2)	*** 1.44 [1.25–1.67]	** 1.27 [1.08–1.48]	*** 1.37 [1.19–1.59]	*** 1.33 [1.14–1.54]	*** 1.37 [1.19–1.57]	*** 1.31 [1.13–1.52]
DRS (≥3 vs <3)	*** 2.05 [1.78–2.36]	*** 1.74 [1.48–2.04]	** 1.26 [1.08–1.48]	* 1.18 [1.00–1.39]	** 1.23 [1.05–1.44]	1.16 [0.99–1.36]
Gender (m vs f)	1.09 [0.96–1.24]		*** 1.35 [1.16–1.56]	*** 1.32 [1.13–1.54]	*** 1.37 [1.19–1.58]	*** 1.32 [1.14–1.53]
Age (years)	1.00 [0.99–1.01]		** 0.99 [0.98–1.00]	** 0.98 [0.97–1.00]	* 0.99 [0.98–1.00]	* 0.99 [0.98–1.00]
Chewing problems						
ADLH (≥3 vs <3)	1.00 [0.86–1.16]		*** 1.91 [1.63–2.23]	*** 1.68 [1.43–1.98]	*** 1.91 [1.64–2.22]	*** 1.67 [1.43–1.96]
CPS2(≥2 vs <2)	*** 1.42 [1.22–1.66]	* 1.22 [1.04–1.44]	*** 2.09 [1.80–2.44]	*** 1.65 [1.41–1.95]	*** 2.08 [1.79–2.41]	*** 1.66 [1.43–1.94]
DRS (≥3 vs <3)	*** 2.04 [1.76–2.36]	*** 1.74 [1.47–2.05]	*** 2.09 [1.79–2.44]	*** 1.88 [1.60–2.21]	*** 2.08 [1.78–2.43]	*** 1.87 [1.59–2.19]
Gender (m vs f)	1.08 [0.95–1.23]		1.11 [0.95–1.30]		1.12 [0.96–1.30]	
Age (years)	1.00 [0.99–1.01]		* 1.01 [1.00–1.02]	1.01 [1.00–1.02]	* 1.01 [1.00–1.02]	* 1.01 [1.00–1.02]
Dry mouth						
ADLH (≥3 vs <3)	1.01 [0.87–1.17]		** 1.23 [1.08–1.40]	** 1.21 [1.06–1.39]	** 1.22 [1.07–1.40]	** 1.20 [1.05–1.38]
CPS2(≥2 vs <2)	*** 1.42 [1.22–1.65]	* 1.23 [1.05–1.44]	1.00[0.88–1.14]		1.00 [0.87–1.16]	
DRS (≥3 vs <3)	*** 1.95 [1.69–2.25]	*** 1.65 [1.40–1.94]	*** 1.89 [1.65–2.17]	*** 1.88 [1.63–2.16]	*** 1.89 [1.65–2.16]	*** 1.87 [1.62–2.14]
Gender (m vs f)	1.10 [0.97–1.25]		* 0.87 [0.75–1.00]	* 0.86 [0.74–1.00]	* 0.86 [0.75–0.99]	* 0.87 [0.75–1.00]
Age (years)	1.00 [0.99–1.01]		1.00 [0.99–1.01]		1.00 [0.99–1.01]	

* p≤0.05

** p≤0.01

*** p≤0.001

Missing values for *dental prosthesis use* occurred significantly more frequent if subjects scored higher on the DRS scale (OR 1.66, CI 1.41–1.95) after correction for CPS2. Missingness was associated with cognitive impairment and depression for *damaged teeth* (CPS2 OR 1.27, CI 1.08–1.48; DRS OR 1.74, CI 1.48–2.04), *chewing problems* (CPS2 OR 1.22, CI 1.04–1.44; DRS OR 1.74, CI 1.47–2.05) and *dry mouth* (CPS2 OR 1.23, CI 1.05–1.44; DRS OR 1.65, CI 1.40–1.94).

Using the complete-case technique, *dental prosthesis use* was significantly associated with better ADLH (OR 0.82, CI 0.73–0.92), female gender (OR 0.73, CI 0.64–0.83) and higher age (OR 1.05, CI 1.04–1.06) after correction for CPS2. *Damaged teeth* were significantly more often registered in subjects with impaired cognition (CPS2 OR 1.33, CI 1.14–1.54) and symptoms of depression (DRS OR 1.18, CI 1.00–1.39). Male gender (OR 1.32, CI 1.13–1.54) and younger age (OR 0.98, CI 0.97–1.00) were also significantly associated to this outcome. *Chewing problems* were significantly associated to poor ADLH (OR 1.68, CI 1.43–1.98), CPS2 (OR 1.65, CI 1.41–1.95), DRS (OR 1.88, CI 1.60–2.21) after correction for age when missing values were treated by the complete-case approach. *Dry mouth* was significantly more often registered in subjects with poor ADLH (OR 1.21, CI 1.06–1.39), DRS (OR 1.88, CI 1.63–2.16) and female gender (OR 0.86, CI 0.74–1.00).

For the four oral health-related variables, after multiple imputation of missing data almost identical odd's ratios—including their 95% confidence intervals—were obtained for the analyzed associations. However, the association between *damaged teeth* and DRS was not significant in the multivariable model after multiple imputation.

## Discussion

In general, three types of missing values are discussed in the literature [22]. If values are *missing completely at random*, the probability of missingness is not related to any observed or unobserved characteristics of the subject. Ignoring this type of missing values does not result in biased statistical results but causes imprecision due to a reduction of statistical power. The *missing not at random* type can be found if the probability of missingness depends on information that is related to unobserved subject characteristics. Universal methods for handling this kind of missing data adequately do not exist and as a result, statistical results are biased. Most frequently, missing values are of the *missing at random* type which means that the probability of missingness may depend on observed characteristics but may not depend on unobserved characteristics of the subject. Statistical methods like multiple imputation provide unbiased statistical results given a correctly specified imputation model [22]. However, missing values are frequently treated by ad-hoc methods like the complete-case technique that is commonly applied by most statistical software packages [2]. Of the 262 epidemiological studies reviewed by Eekhout et al. (2012), 5% provided information on item missingness. The reported levels ranged from 1% to 44% [2], which corresponds to the findings of the present study with missing values ranging from 16.3% to 17.2% for the individual oral health-related variables. According to the analytic guidelines of the National Health and Nutrition Examination Survey in the United States, further examination of non-respondents with respect to the outcome is necessary if more than 10% of the data for a variable are missing [23].

In our study, as hypothesized, missingness in all oral health-related variables was significantly associated with the occurrence of symptoms of depression. The odds for missingness were about 1.7 times higher in depressed individuals. Except for *dental prosthesis use*, missingness was also related to cognitive impairment with approximately 1.2 times higher odds of missing oral health-related information. Opposing our hypothesis, ADLH was not associated with missingness. The presented results indicate that the prevalence of missing data in the oral health-related variables is not completely at random and needs further investigation.

Literature on the causes of item-missingness in epidemiological research is scarce. A study with community-residing older individuals revealed that missing values of the items *employment* and *finances* in a personal interview were associated with self-rated physical health, mental health, and cognitive functioning [24]. In accordance with the results of our study, missingness was significantly more pronounced in subjects with poor mental health and cognitive functioning. For self-rated physical health no significant group differences were found [24]. Based on the interRAI-HC, Vanneste et al. (2015) found the highest amounts of missing data in the predominantly medically-oriented items, including disease diagnoses and drug allergies. The authors suggest that this might be caused by the fact that the nurses who performed the assessment had only limited access to this information in the home-care environment. Items that required a careful and thorough inspection of the client were also completed less thoroughly [4].

During the interRAI-HC assessment, oral health-related data are collected by observation, interview and/or inspection of the mouth. Hence, we can only speculate about the mechanisms responsible for the associations found in our study. A poor cognitive status of the subject might have an impact on cooperation and the ability to process and answer questions of the interRAI-HC. Attitudes of general indifference and disinterest, inherently connected to a depressive disorder, might also affect cooperation during the interRAI-HC. Moreover, assessing oral health in cognitively impaired and depressed individuals may be more challenging for the assessors, who are usually not trained as oral health professionals. The finding that ADLH was not associated with missingness might be attributed to the fact that a poor functional condition does not imply a reduction in cooperative behavior. Furthermore, a frail functional status might evoke more empathy, leading to a more precise assessment.

In our study, multiple imputation was applied to evaluate the impact of item missingness on statistical results concerning the associations between oral health and selected variables of general health. The well-founded multiple imputation technique estimates missing data by using available information of the subjects. Multiple data sets are created with different imputations, based on a random sample from different estimated underlying distributions. After imputation, analysis is performed with each imputed dataset and the resulting estimates and their corresponding standard errors are pooled. As this approach accounts for the various aspects of uncertainty of the imputations, it results in statistically valid estimates [22]. A review based on 262 studies, published in one of the three leading epidemiology journals, revealed that only 8% had applied multiple imputation. In contrast, 81% of the studies had used the complete-case procedure, completely ignoring the potential bias introduced by missing data [2].

In our study, *dental prosthesis use* was significantly associated with a better ADLH status. The relationship between denture wearing and activities of daily life was confirmed by Furuta et al. (2013) [25]. In line with international literature [7–10, 12], the current study found oral health-related problems associated with an inferior status in other health areas. Contradicting our second hypothesis, all associations between oral health-related variables and predictors were characterized by nearly identical odd's ratios before and after multiple imputation. Almost complete overlap of the corresponding 95% confidence intervals confirmed that statistical results did not differ between the two approaches. This finding might be explained by the fact that associations between missing data and predictors were too small to largely bias statistical results based on the whole dataset. To examine the impact of the statistical procedure used, we also investigated risk ratios by using a log-link. This approach lead to similar results.

However, on a subject level, missing data in the interRAI-HC implies the potential under-detection of a condition that requires care. Comparing the frequencies of oral health-related problems before and after multiple imputation, 0.28%, 0.20% and 0.10% of all subjects with *damaged teeth*, *chewing problems and dry mouth* were not registered as having these problems (Table 1). Given the extensive literature on the interaction between oral and general health [7–10, 12], further research should concentrate on the mechanisms that mediate the occurrence of missing data to develop preventative strategies.

It is a limitation of the present study that the sample is not representative for all older individuals receiving home care in Belgium. Furthermore, validity of the oral health-related variables of the interRAI needs to be considered. Folse (2001) reported a profound under-detection of oral health-related problems after comparing interRAI data with dental examination forms [26]. A similar tendency was described by Nordenram et al. (2002) based on oral examination data and interRAI assessments from the same subjects [9]. An international study on inter-rater reliability also revealed that, compared to other items of the interRAI, the oral health-related items had relatively low mean kappa values (between 0.6 and 0.7) [1].

However, the above mentioned papers concerned an earlier version of the interRAI than the one that was used in this study. Further research is necessary in order to clarify, if processes that mediate the occurrence of missing data are linked to problems of validity and reliability.

International extrapolation of our results has to consider the context of the Belgian health care system, where a complete interRAI assessment was not required for coverage of care. If this is the case, arbitrary responses or automatic response patterns might replace missing data problems.

## Conclusion

Data revealed that cognitively impaired and depressive individuals had a higher risk of oral health-related problems and missing oral health-related data. Associations between oral health and ADLH, CPS2 and DRS were not influenced by multiple imputation of missing data.
